# Hepatocytic parental progenitor cells of rat small hepatocytes maintain self-renewal capability after long-term culture

**DOI:** 10.1038/srep46177

**Published:** 2017-04-11

**Authors:** Masayuki Ishii, Junichi Kino, Norihisa Ichinohe, Naoki Tanimizu, Takafumi Ninomiya, Hiromu Suzuki, Toru Mizuguchi, Koichi Hirata, Toshihiro Mitaka

**Affiliations:** 1 Department of Tissue Development and Regeneration, Research Institute for Frontier Medicine, Sapporo Medical University School of Medicine, South-1, West-17, Chuo-ku, Sapporo 060-8556, Japan; 2 Department of Surgery, Surgical Oncology and Science, Sapporo Medical University School of Medicine, Sapporo Medical University School of Medicine, South-1, West-17, Chuo-ku, Sapporo 060-8543, Japan; 3 Tokushima Research Institute, Otsuka Pharmaceutical Co. Ltd., 463-10 Kagasuno, Kawauchi-cho, Tokushima 771-0192, Japan; 4 Department of Anatomy I, Sapporo Medical University School of Medicine, South-1, West-17, Chuo-ku, Sapporo 060-8556, Japan; 5 Department of Molecular Biology, Sapporo Medical University School of Medicine, South-1, West-17, Chuo-ku, Sapporo 060-8556, Japan

## Abstract

The liver has a variety of functions for maintaining homeostasis, and hepatocytes play a major role. In contrast with the high regenerative capacity of mature hepatocytes (MHs) *in vivo*, they have not been successfully expanded *ex vivo*. Here we demonstrate that CD44-positive cells sorted from small hepatocyte (SH) colonies derived from a healthy adult rat liver can proliferate on a Matrigel-coated dish in serum-free chemically defined medium; in addition, a subpopulation of the cells can divide more than 50 times in a period of 17 weeks every 4-week-passage. The passage cells retained the capability to recover highly differentiated functions, such as glycogen storage, CYP activity and bile secretion. When Matrigel-treated cells from the third passage were transplanted into retrorsine/partial hepatectomy-treated rat livers, the cells engrafted to differentiate into MHs and cholangiocytes. These results suggest that long-term cultured CD44^+^ SHs retain hepatocytic characteristics *in vitro* and the capability to differentiate into hepatocytes and cholangiocytes *in vivo*. Thus, a newly identified subpopulation of MHs possessing the attributes of hepatocytic stem/progenitor cells can be passaged several times without losing hepatocytic characteristics.

The liver performs various functions, such as the regulation of energy homeostasis, biosynthesis of plasma proteins, excretion of endogenous metabolic end-products and xenobiotics, and bile production and secretion^1^,^2^. These functions are obviously essential for an animal’s life; thus, the liver has a great capacity for regeneration against hepatic damages to maintain homeostasis. When animals, including humans, suffer from a severe liver injury, specific metabolic deficiency, or end-stage liver disease, the proliferative capability of liver cells is insufficient to compensate for the loss of function. In diseased patients, a whole or segmental liver transplantation is widely chosen as the last option to save their lives. However, this possibility is limited by a persistent shortage of donor organs. Cell-based therapies, such as cell transplantation, engineered hepatocellular tissue constructs, and bio-artificial liver devices, may be used as alternatives to whole liver transplantation^3^,^4^,^5^. These therapies require a large number of healthy hepatocytes, but it is currently not feasible to routinely obtain healthy human hepatocytes because of severe liver donor shortages and a lack of methods to generate functional hepatocytes.

Hepatocytes are the predominant cell type in the liver, constituting over 80% of the liver mass under physiological conditions^1^. Unlike the gastrointestinal tract or hematopoietic system, in which a continual loss of mature cells is consistently replenished by stem-cell-driven cell production, the healthy liver is essentially a stable population containing only occasional hepatocytes that enter the cell cycle in response to “wear and tear” cell loss. However, the liver is capable of regenerating after surgical resection or cytotoxic damage. Following a 2/3 partial hepatectomy (PH), the residual cells proliferate and replace lost liver mass^6^. Serial PH revealed that rat mature hepatocytes (MHs) were capable of at least 18 cell doublings^7^. On the other hand, serial transplantations of mouse hepatocytes to immune-deficient mouse livers with fatal liver disease showed that hepatocytes possess tremendous replication potential^8^,^9^. However, it is well known that primary hepatocytes exhibit poor growth activity *in vitro* while maintaining metabolic and xenobiotic functions^5^,^10^. The lack of appropriate culture protocols for expanding hepatocytes is a bottleneck for their application. Only a few studies have reported the multiplication of primary hepatocytes with differentiated functions. These procedures are divided into two major methods: the use of a high concentration of nicotinamide^11^,^12^,^13^ and a chemically defined nutrient-rich medium supplemented with growth factors and DMSO^14^,^15^. Under these culture conditions, the number of cells increased, at most, two- to three-fold. To overcome the growth limitations of primary human hepatocytes, human hepatocyte cell lines were generated by the introduction of immortalized genes^16^,^17^. Recently, improved culture systems of human hepatocytes were reported. Human primary hepatocytes cocultured with embryonic fibroblasts could expand with the addition of small molecules^18^. Oncostatin M-dependent human hepatocytes introduced through human papilloma genes could also expand in medium containing serum^19^. Although these cells possess the capability to proliferate and regain differentiated hepatic functions, they depend on serum and feeder cells or require genetically manipulation. When generating hepatocytes for cell-based therapies, cells with as few modifications as possible is preferable, and the cells must be cultured in a chemically defined medium containing no animal-derived materials, such as serum. Therefore, the establishment of a more ideal culture method for hepatocyte expansion is urgently needed.

Various attempts have been made over the last several decades to harness the innate replication potential of hepatocytes *ex vivo*. The highest growth potential of liver cells in culture has been attributed to a hepatocyte subpopulation called small hepatocytes (SHs)^20^,^21^. Rat and human SHs isolated from a healthy adult liver proliferated to form colonies in a serum-free medium on hyaluronic acid (HA)-coated dishes^22^,^23^, which may be dependent on CD44 expression at the cell surface^24^. The colonies originated from a single cell and consisted of approximately 30–40 cells after 8–10 days of plating. Cell colonies morphologically showed a polygonal shape similar to MHs but differed in size and level of attachment between cells. While the size and other features of most colonies were uniform in the early culture period, some SHs spontaneously redifferentiated into MHs^25^,^26^. SH maturation was induced by Matrigel treatment^27^,^28^. However, a population of cells with small-sized morphology and the ability to proliferate is consistently maintained in culture. Therefore, we speculate the existence of parental SHs, which are true hepatocyte progenitors, in adult livers.

In the present study, we confirmed the existence of hepatocytic progenitor cells within SHs. These progenitors were isolated as CD44^+^ cells from SH colonies. These cells retained their expression of albumin, HNF4α, and CD44, and possessed self-renewal capability for up to 17 weeks after isolation. Furthermore, these cells maintained the ability to recover highly differentiated functions and re-establish a network of bile canaliculi (BCs) when overlaid on Matrigel. The transplantation of Matrigel-treated third passage cells to retrorsine/PH (Ret/PH)-treated rat livers showed that donor cells engrafted in the liver and proliferated to form foci. In addition, we observed that some cells incorporated into small bile ductules and differentiated into cholangiocytes. Thus, long-term cultured CD44^+^ SHs retain hepatocytic characteristics *in vitro* and the capability to differentiate into hepatocytes and cholangiocytes *in vivo*.

## Results

### Efficient subculture of small hepatocytes on Matrigel

**Figure 1:**
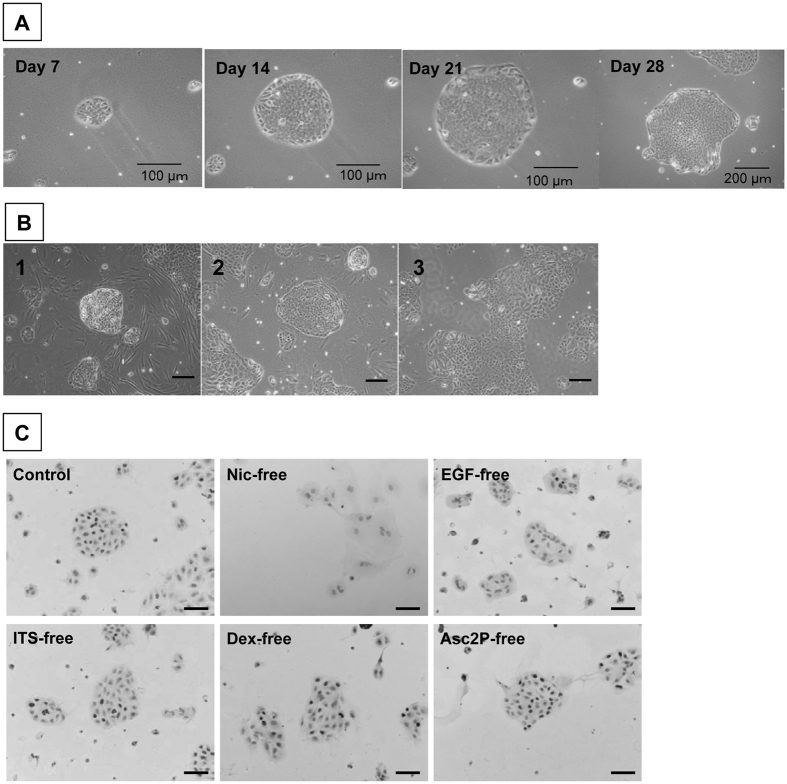
Growth of sorted CD44^+^ SHs on a Matrigel-coated dish. (**A**) Phase-contrast photos of first passaged CD44^**+**^ SHs formed typical colonies and showed high growth activity. The same colony, derived from a single cell, was followed until day 28. Scale bars = 100 μm (Days 7, 14, and 21) and 200 μm (Day 28). (**B**) Typical features of colonies observed in the culture of CD44^**+**^ SHs at 28 days after the first passage. (**C**) The necessity of constituents in the culture medium for the growth of CD44^+^ SHs. Immunocytochemistry for BrdU in cells separately cultured in medium lacking each constituent at 14 days after plating. The control shows the colony formation of cells in the medium with all constituents. Cells were cultured in medium lacking nicotinamide (Nic-free), epidermal growth factor (EGF-free), ITS-free, dexamethasone (Dex-free), or ascorbic acid 2-phosphate (Asc2P-free). BrdU^+^ nuclei are shown in black and nuclei were counterstained with hematoxylin (Gray). Scale bars = 100 μm.

**Figure 2:**
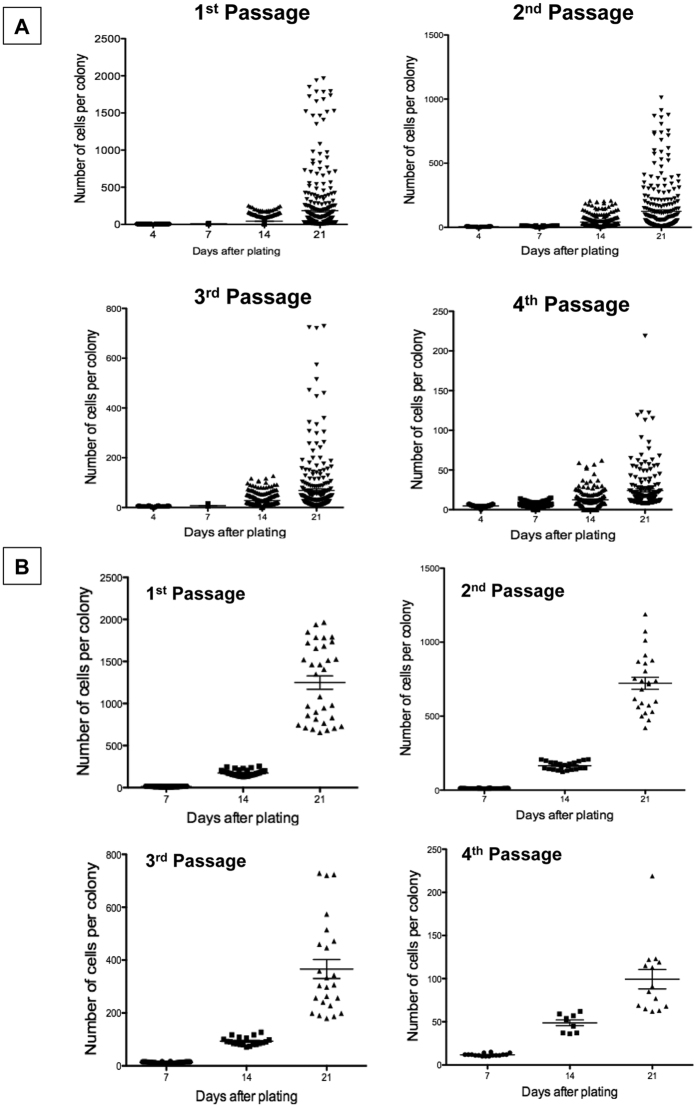
Growth of passaged cells. (**A**) The colony-size distribution is shown for each passage at days 4, 7, 14, and 21 after plating. The number of cells per colony for all colonies from 3 dishes was measured. (**B**) The data shown in A that included cells within the upper 10% of colonies were replotted. The number of colonies is shown in Table 1.

**Figure 3:**
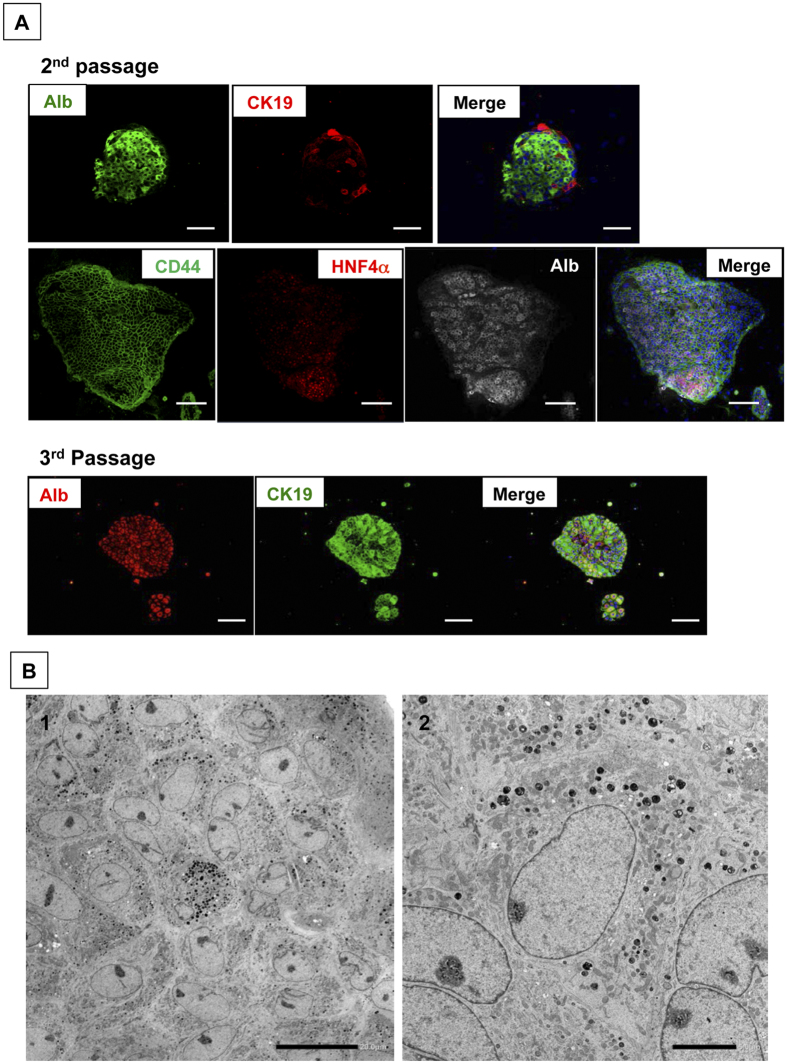
The characteristics of passaged colony-forming cells. (**A**) Double and triple fluorescent immunocytochemistry for albumin/CK19 and CD44/HNF4α/albumin, respectively, was performed in second and third passage cells at 28 days after plating. Scale bars = 100 μm. (**B**) Transmission electron micrographs of second passage cells at 14 days after plating. Scale bars = 20 μm (B-1) and 5 μm (B-2).

Primary SHs isolated from a healthy adult liver proliferated to form colonies in serum-free medium on HA-coated dishes as previously reported^22^. To subculture SHs, we previously showed that colony isolation enables SHs to maintain their proliferative capability even after cryopreservation^28^. However, when we dissociated SH colonies into single cells using trypsin, a small number of cells attached to the collagen type I-coated dish but did not proliferate. To identify parental SH cells, it is necessary to prove that the cells can clonally proliferate. Therefore, we initially explored the conditions in which a single SH separated from colonies could proliferate. To obtain the purest SHs possible, the cells were cultured on HA-coated dishes in serum-free medium for 9 days. As trypsinization may injure cells, we chose to detach colonies using collagenase and hyaluronidase, and the colonies were broken into single cells by rigorous stirring. Next, we sorted SHs using anti-CD44 antibodies and MACS because most cells in SH colonies are CD44^+ ^24. We assessed if sorted CD44^+^ SHs grow to form colonies. The sorted cells did not expand into colonies on collagen type I-coated or HA-coated dishes. Notably, CD44^+^ SHs actively proliferated on Matrigel-coated dishes (Fig. 1A). A Matrigel concentration between 0.2 and 2.0 mg protein/mL did not affect the efficiency of colony formation (Supplemental Figure S1). The efficiency of cell attachment on Matrigel-coated dishes and colony formation was 16.9 ± 0.5% (Supplemental Table S3) and approximately 0.12% (approximately 1,800 colonies/150,000 cells) of plated CD44^+^ SHs, respectively. Although the features varied, epithelial colonies showed a cell morphology typical of hepatocytes, including a polygonal shape, dense cytoplasm, and bright round nucleus (Fig. 1B). Binucleate cells were observed, but cells with multinuclei were rarely detected. In addition, irregular shaped colonies were often composed of relatively large cells or the combination of small- and large-sized cells (Fig. 1B-3). Supplemental Figure S2 and Supplemental Table S4 show that approximately 52% and 44% of first passage cells cultured for 4 weeks are diploid and tetraploid, respectively. Octaploid cells represented approximately 4% of the population. In addition, the ratios of mono- and bi-nuclear cells in colonies at 14 days were 93.2 ± 3.6% and 6.5 ± 3.6%, respectively.

To examine which constituents of the medium are necessary for CD44^+^ SHs to proliferate and form colonies, the cells were cultured in separate mediums lacking each component. As shown in Fig. 1C and Supplemental Table S5, nicotinamide was indispensable for the formation of colonies. In addition, EGF, insulin, and Dex enhanced the proliferation of cells.

CD44^+^ SHs on Matrigel-coated dishes were capable of expanding for more than 4 weeks. To investigate whether CD44^+^ SHs could continue to grow while maintaining their colony-forming capacity, a second passage was performed 4 weeks after the first passage. The dissociated cells were plated on new Matrigel-coated dishes, and passages were repeated every 4 weeks (Supplemental Figure S3). A portion of passaged cells attached to the dish and proliferated to form colonies. The rates of cell attachment and expansion for each passage are shown in Supplemental Table S3. Approximately 15% of the plated cells attached to the Matrigel-coated dishes, and this rate gradually decreased with increasing passages. The attached cells proliferated to form colonies, and the total number of cells at day 28 showed an approximate 12-, 11-, 4-, and 4-fold increase when compared with cells at day 1 in the first, second, third, and fourth passages, respectively.

### Possible parental cells for small hepatocytes show extremely high proliferative capability

Supplemental Figure S4 indicates the morphology of typical colonies observed in each passage. Colonies showing a high growth potential possessed a relatively round shape, which consisted of homogeneously small-sized, polygonal, and mononuclear cells (Supplemental Figs S4–1–3). Colonies with irregular shapes, which consisted of cells with a relatively large cytoplasm (Supplemental Figs S4–4–9), appeared in the cultures over time. The number of cells per colony at days 4, 7, 14, and 21 was counted at each passage, and the size distribution is shown in Fig. 2A. The average size of the colonies at 21 days after passage was 218.1 ± 429.6, 149.2 ± 233.1, 83.7 ± 139.9, and 32.9 ± 45.9 cells/colony in the first, second, third, and fourth passages, respectively. The distribution of colony sizes indicates that a population of cells with a high growth potential exists in each passage, although this population decreases with increasing passages. We verified that the distribution of colony sizes with a high growth potential was not different among experiments until the second passage, i.e., differences were observed after the third passage (Supplemental Figure S5). Therefore, we selected the colonies with a high growth potential (upper 10% of colonies each day) for each passage, and their size distribution was replotted (Fig. 2B). Compared to the average number of cells shown in Fig. 2A, the number of colonies at 21 days jumped up to 1249.2 ± 454.5, 722.3 ± 198.5, 366.2 ± 176.1, and 99.3 ± 41.8 cells/colony in the first, second, third, and fourth passages, respectively. To estimate the number of cell divisions, the formula of exponential approximation was calculated from the data shown in Fig. 2B (Supplemental Figure S6A and Table 1). To establish linear equations for the first, second, third, and fourth passages, the mathematical expression was converted to a logarithmic scale (Supplemental Figure S6B and Table 1), and the average number of cell divisions over a period of 28 days was estimated for each passage. The estimated number of cell divisions was 13.5, 12.7, 11.0, and 9.1 in the first, second, third, and fourth passages, respectively. Many primary SH colonies consisted of >32 cells/colony at 9 days; thus, we determined the duplication number of primary SHs with a high growth capability was 6. Based on these results, CD44^+^ SHs with a high growth potential divided more than 52 times over 17 weeks. We hereafter refer to cells with a high growth potential as hepatocytic parental progenitor cells (**HPPCs**).

### HPPCs have the characteristics of bipotential hepatic stem cells

To determine if proliferating cells are derived from hepatocytes, we investigated the characteristics of cells within the colonies. As shown in Fig. 3A, immunocytochemical staining shows that most colonies consist of albumin-positive cells. Although CK19^+^ cells were found in every colony, the ratio of these cells varied and increased with each passage. Regardless of the number of passages, most cells in the colonies maintained CD44-positivity. Hepatocyte-enriched transcription factors, such as HNF4α and C/EBPα, were expressed in the nucleus of many cells until the second passage (Fig. 6B). While HNF4α expression was maintained in most cells at the fourth passage, C/EBPα^+^ cells were rarely observed in third and fourth passage cells. The immunocytochemical findings were confirmed by qPCR (Supplemental Figure S7). The ultrastructure of second passage cells indicated that small-sized cells with rich organelles, such as mitochondria, rER, and the Golgi apparatus, are tightly conjunct (Fig. 3B). In addition, they possessed glycogen granules in their cytoplasm. Bile canaliculi were not apparent between cells.

**Figure 4:**
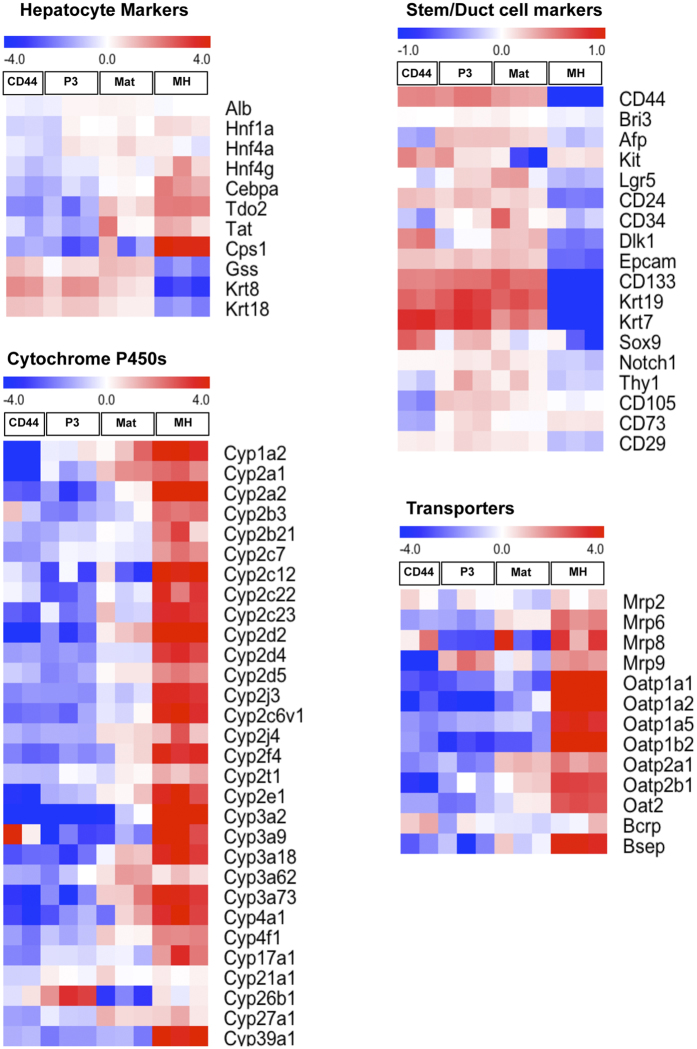
Comprehensive analysis of gene expression of third passage cells. An oligo microarray was used for analysis. After a 28-day culture of second passage cells, total RNA was extracted from trypsinized cells. Representative genes were selected to show heat maps. Specific markers are divided into four groups, including hepatocyte-specific, cytochrome P450s, stem/duct cell-related markers and hepatic transporters. CD44, CD44-positive cells sorted from primary SH colonies; P3, third passage cells; Mat, third passage cells treated with Matrigel-overlay for a week after 14-day culture of second passage cells; MH, mature hepatocytes. To obtain third passage cells, second passage cells were cultured for 28 days with or without Matrigel-overlay and then dissociated cells were collected.

A comprehensive analysis of the gene expression in third passage cells was examined using DNA microarrays. As shown in Fig. 4, genes related to higher differentiated functions, including *Cebpa, Tdo2, Tat, Cyps*, and transporters were scarcely expressed in passaged cells (P3). However, the cells expressed many hepatocyte markers, including *Alb, Hnf4α*, glutamine synthetase (*Gss*) and Keratins (*Krt*) 8 & 18. The cells also expressed stem/duct cell markers, including *Afp, Lgr5, CD24, CD133, Epcam, Sox9, Krt 7*, and *Krt19*.

### HPPCs redifferentiate into functional hepatocytes *in vitro*

**Figure 5:**
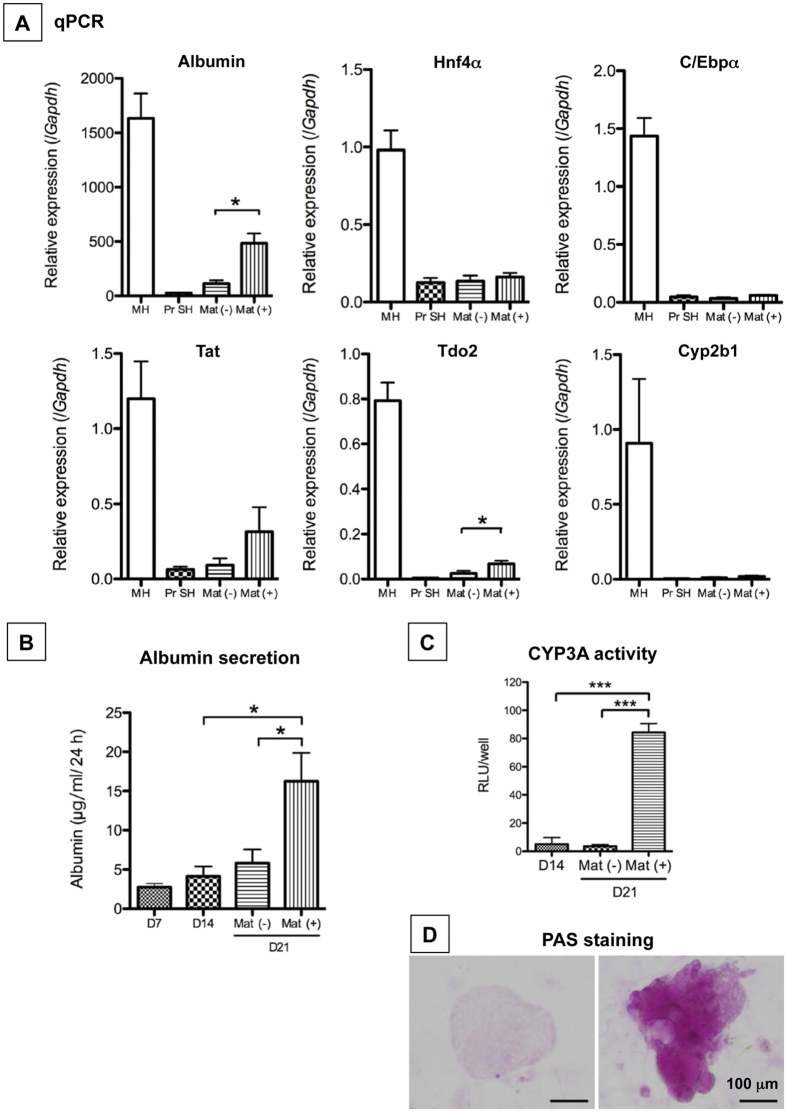
Capability of maturation of second passage cells grown with Matrigel. Second passage cells were cultured for 14 days and then treated with Matrigel for 7 days. (**A**) The gene expression of *Alb, Hnf4a, Cebpa, Tat, Tdo2*, and *Cyp2b1* was measured by qPCR. The second passage cells were cultured for 21 days [Mat(−)], and a subset of cells was treated with Matrigel from day 14 to day 21 [Mat(+)]. MH: isolated hepatocytes from a normal adult liver; PrSH: CD44^+^ SH. *p < 0.05. (**B**) Albumin secretion by second passage cells was measured using ELISA. The second passage cells were cultured for 21 days [Mat(−)], and a subset of cells was treated with Matrigel from day 14 to day 21 [Mat( + )]. *p < 0.05. (**C**) CYP3A activity was measured in second passage cells treated without [Mat(−)] or with Matrigel [Mat(+)]. *p < 0.005. (**D**) To examine the ability of cells to accumulate glycogen, PAS staining was performed in cells without [Mat(−)] or with Matrigel [Mat(+)]. Scale bar = 100 μm.

To determine if passaged cells have the capacity to redifferentiate into MHs, cells were treated with Matrigel for 1 week. A comprehensive gene expression analysis revealed that the cells treated with Matrigel (Mat) partially recovered the expression of genes related to highly differentiated hepatic functions, including *Tdo2, Tat, Cyps,* and *Oatp2*; however, the expression level was lower than that of MHs (Fig. 4). This result was confirmed by qPCR. The expression of *Alb, Hnf4a, Cebpa, Tat, Tdo2,* and *Cyp2b1* in cells treated with Matrigel was apparently lower than in MHs. However, expression of *Alb* and *Tdo2* was significantly increased compared to the cells not treated with Matrigel (Fig. 5A). In addition, the secretion of albumin into the culture medium was increased (Fig. 5B) and CYP3A activity was markedly induced (Fig. 5C). PAS staining demonstrated that glycogen accumulated in the cytoplasm of cells treated with Matrigel (Fig. 5D).

**Figure 6:**
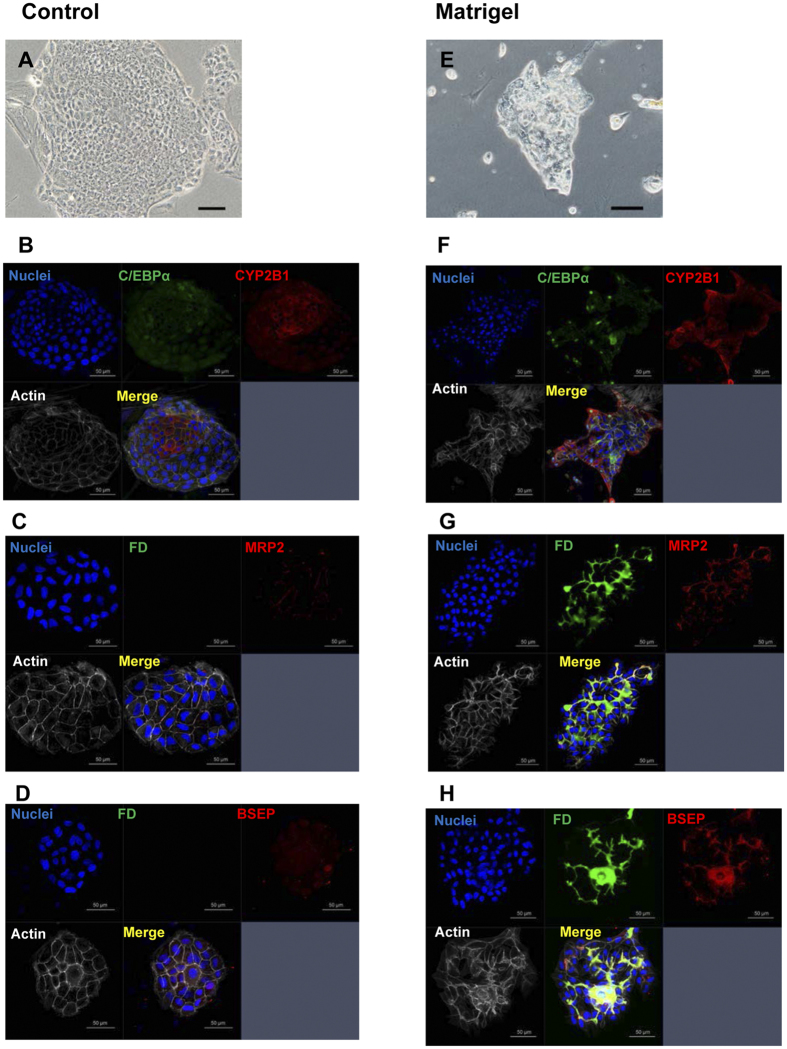
Bile canaliculi formation of second passage cells. The cells were cultured for 21 days (Control; **A**–**D**), and a subset of cells was treated with Matrigel from day 14 to day 21 (Matrigel; **E**–**H**). Phase-contrast photos show a typical colony with (**E**) or without Matrigel-treatment (**A**). Fluorescent immunocytochemistry for C/EBPα/CYP2B1 was conducted (**B** and **F**). Fluorescent images were taken soon after the addition of fluorescein diacetate (FD). Cells were fixed and fluorescent immunocytochemistry was performed (**C**,**D**,**G**, and **H**). BC formation was verified with MRP2/BSEP/actin expression (**C**,**D**,**G**, and **H**).

We previously reported that SHs reconstructed hepatic organoids with BC-networks^25^. To investigate if HPPCs formed BC-networks, fluorescein diacetate (FD) was administered to HPPCs treated with Matrigel. As illustrated in Fig. 6, a control colony of HPPCs showed a monolayer of small-sized flattened cells, whereas the colony treated with Matrigel showed relatively large cells that piled on top of each other to form a 3D structure. Fluorescein secreted into BCs suggested the formation of fine networks, and the dye accumulated in some cystic structures. Apical membrane proteins, such as MRP2 and BSEP, were expressed along the BCs, and the structure was lined with actin fibers.

### Transplanted HPPCs repopulate recipient liver tissue

**Figure 7:**
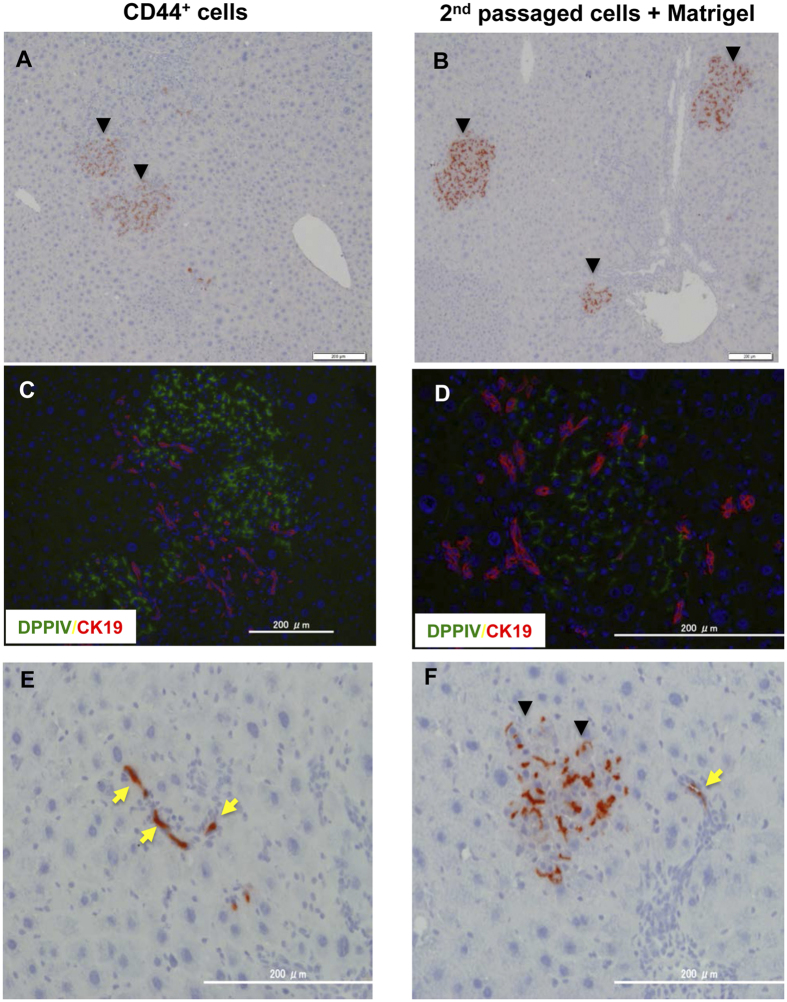
Repopulation of DPPIV^+^ donor cells in Ret/PH-treated DPPIV^−^ rat livers. Primary SHs were cultured for 9 days, and cells from SH colonies were isolated using an anti-CD44 antibody (CD44-SHs; **A**,**C**, and **E**). The second passage cells were cultured for 14 days and then treated with Matrigel for 7 days. The cells were detached by trypsin, and separated cells were transplanted (2^nd^ passaged cells + Matrigel; **B**,**D**, and **F**). At 30 days after transplantation, livers were removed and fixed. Enzyme histochemistry for DPPIV is shown in **A**,**B**,**E** and **F**. Fluorescent immunohistochemistry for DPPIV and CK19 was performed (**C**,**D**). The arrowheads in **A**,**B**, and **F** indicate DPPIV^+^ foci derived from donor cells. Yellow arrows in **E** and **F** show DPPIV^+^ donor cells incorporated into small bile ductules.

To confirm that HPPCs can differentiate into MHs *in vivo*, we first transplanted separated DPPIV^+^ cells after the third passage into DPPIV^−^ female rat livers treated with Ret/PH via the spleen. Although the CD44^+^ SHs engrafted in liver lobules to form DPPIV^+^ foci (Fig. 7A and C), the third passage cells did not engraft in Ret/PH rat livers. However, second passage cells treated with Matrigel for 1 week and then transplanted into Ret/PH rat livers showed many DPPIV^+^ foci (Fig. 7B and D). The donor cells engrafted in all of the rat livers (5/5). Although few in number, we also observed that both CD44^+^ cells and third passage cells with Matrigel were present in the small bile ductules (Fig. 7E and F and Supplemental Figure S8).

## Discussion

We previously reported that SHs are a subpopulation of MHs and possess the potential to be hepatocytic progenitor cells^10^,^29^. Importantly, SHs can be obtained from a healthy adult liver, and more than 1% of MHs have the potential to be SHs^30^. Our present study reveals that HPPCs exist among CD44^+^ SHs and continue to generate daughter cells for several months. These cells retain the capability of proliferation and redifferentiation but gradually lose self-renewal ability. Rat HPPCs have the potential to divide more than 50 times in a period of 17 weeks through four passages. Given the limited number of human healthy hepatocytes, it is important to establish an expansion method of hepatocytes for research and clinical use. In this study, we expanded progenitor cells possessing hepatocytic characteristics derived from healthy adult rat livers using chemically defined culture conditions.

The tremendous *in vivo* growth ability of MHs, especially from mice, has been reported. Serial transplantations of mouse hepatocytes into fumarylacetoacetate hydrolase (FAH)-deficient mice showed that these cells underwent more than 70 cell doublings after seven rounds of transplantation^8^. Wang *et al*.^9^ recently reported that hepatocytes isolated from aged mouse livers had the capacity to repopulate the recipient liver after 12 rounds of serial transplantation using a similar method. These results indicate that MHs or their subpopulation have the potential for continuous proliferation. In contrast, the growth ability of MHs is limited *in vitro*. Shan *et al*.^18^ reported on a human liver hepatocyte culture system with only a 10-fold expansion over a period of 1 week. Oncostatin M-dependent expansion of primary human hepatocytes introduced through human papilloma genes underwent up to 40 population doublings^19^. Although these human cells possess the capacity to proliferate and regain hepatic differentiated functions, they require serum, feeder cells, or genetic manipulation for their proliferation, which are not suitable culture conditions for clinical use. Rat HPPCs, however, can expand in simple culture conditions. The cells are cultured on Matrigel-coated dishes in a chemically defined serum-free medium with growth factors and a high concentration of nicotinamide, which is essential for their growth. Recently, 5–10 mM nicotinamide has been routinely added to media for stem/progenitor cells, including pluripotent stem cells^31^,^32^,^33^. Although the detailed mechanism remains unclear, nicotinamide may be necessary to induce the progenitor potential of these cells. However, HPPCs gradually lose the ability to generate daughter cells and ultimately die. We did not observe cell immortalization; thus, an HPPC clone has not been established. To our knowledge, a clone derived from human hepatocyte has never been generated without gene transfection. Notably, we recently reported that clones of SHs isolated from a healthy adult mouse liver were established, and these clones retain the potential to differentiate into MHs *in vitro* and *in vivo*^34^. These findings indicate that differences in self-renewal potential exist between animals.

Approximately, 1–2 × 10^7^ CD44^+^ SHs were obtained from an 8-week-old rat liver. The plating efficiency of the cells on Matrigel-coated dishes was approximately 0.17, and most attached cells continued to proliferate for 28 days (first passage cells). At the time of the second passage, the total number of cells reached approximately 3.1 × 10^7^ (1.5 × 10^7^ × 0.17 × 12). The efficiency of attachment and the rate of expansion of the second passage cells were approximately 0.15 and 11-fold, respectively. Eight weeks after the first passage, primary CD44^+^ SHs increased to approximately 5.1 × 10^7^ cells (1.5 × 10^7^ × 0.17 × 12 × 0.15 × 11). The rate of the attached cells on Matrigel was consistently approximately 15% of passaged cells, and the rate of colonies with high growth ability was approximately 10% of the total formed colonies in each passage. However, some attached cells may be HPPCs that are unidentifiable with specific markers. The number of HPPCs gradually decreased with passage, whereas HPPCs retained the ability to generate daughter cells.

During the subculture of CD44^+^ SHs, two morphologically distinguishable colonies emerged. One type included round-shaped colonies consisting of homogeneously small-sized mononuclear diploid cells. The other colonies showed various morphologies and consisted of large cells, binuclear cells, and/or tetraploid cells. The number of cells possessing triple nuclei was quite small, and the rate of octaploidy in passaged cells was much smaller than that of primary CD44^+^ SHs. Interestingly, despite the phenotypical alterations among colonies, colony-forming cells expressed hepatocytic genes, including albumin and HNF4α, though cells that formed round-shaped colonies showed a tendency toward a higher expression of these genes when compared with daughter cells. Thus, we speculate that small cells forming round-shaped colonies were HPPCs, whereas the other colonies consisted of daughter cells. This classification is supported by additional experiments. After Matrigel-overlay to induce differentiation, colonies consisting of HPPCs reconstructed hepatic organoids with BC-networks much better than colonies consisting of daughter cells. Although second passage cells treated with Matrigel repopulated the recipient liver tissue, repopulation was not as efficient as primary CD44^+^ SHs. This low efficiency may correlate with the fact that only HPPCs, representing approximately 15% of colonies, maintain their hepatocytic redifferentiation potential. These results suggest that it is necessary to expand the number of HPPCs to generate a large number of hepatocytes with highly differentiated functions. Thus, advanced methods that address the following issues are needed: (1) improve the technique for isolating SHs; (2) increase the number of passaged cells that attach to Matrigel-coated dishes; (3) increase the number of HPPCs; (4) increase the growth rate of HPPCs; (5) prevent the senescence of HPPCs; and (6) improve the method for maturation, particularly without using Matrigel.

The localization of HPPCs remains uncertain because a specific cell marker has yet to be created. Hepatocytes contain heterogeneous cell populations showing different metabolic functions and proliferative capabilities. The liver lobule is segregated into three areas along the porto-central vein axis, namely Zones 1, 2, and 3, and hepatocytes in each zone show distinct expression patterns of metabolism-related genes^35^. A previous report determined that pericentral hepatocytes proliferated slightly faster than those in other areas^36^. Recently, Axin2^+^ pericentral hepatocytes were reported as physiological progenitors for hepatocytes during cellular turnover in the normal mouse liver^37^. Conversely, we reported that Sox9^+^ EpCAM^−^ biphenotypic hepatocytes derived from MHs emerged adjacent to expanding ductular structures in a 3,5-diethoxycarbonyl-1,4-dihydrocollidine-fed mouse liver^38^. Sox9^+^ periportal hepatocytes were also identified as hepatocyte progenitors during regeneration from chronic liver injury^39^. Furthermore, we showed that small mononuclear hepatocyte progenitors existed in the ICAM-1^+^ fractions isolated from healthy adult mouse livers^34^. The ICAM^+^ HNF4α^+^ mononuclear cells did not specifically localize in liver lobules and showed a low expression of both Axin2 and Lgr5 genes. HPPCs expressed Lgr5, whereas Axin2 gene expression was much lower than that of MHs (Fig. 4). The size-dependent growth potential of MHs *in vivo* was investigated by measuring the repopulation capacity after cell transplantation into rodent model livers. Katayama *et al*.^40^ showed that small-sized rat hepatocytes transplanted into Ret/PH-treated rat livers proliferated faster than large-sized cells, whereas Overturf *et al*.^41^ reported that small-sized mouse hepatocytes transplanted into FAH-deficient mouse livers had a lower repopulation capacity when compared with medium- and large-sized cells. Although rat HPPCs and mouse ICAM^+^ HNF4α^+^ cells are small, both HPPCs treated with Matrigel and ICAM^+^ HNF4α^+^ cells treated with OSM, which may enlarge the cells, showed higher efficiency at engrafting and repopulating recipient livers^34^. These accumulated findings suggest that MHs have at least one feature of stem/progenitor cells: long-term renewal and redifferentiation capabilities. Whether or not a single subpopulation of MHs possesses progenitor potential and the location of these cells remains to be determined.

SH colonies efficiently emerge on HA-coated dishes. CD44, a receptor for hyaluronic acid, is consistently and specifically expressed in SHs. However, HPPCs expand on Matrigel and not HA, suggesting that the ECM components in Matrigel are crucial for HPPCs to maintain their proliferation and differentiation capabilities. Although CD44 is not expressed in MHs or early cultures of SHs, the expression becomes apparent soon after a distinct colony is formed^24^. Although primary SHs need HA to attach and grow in serum-free conditions, most passaged cells did not attach to HA-coated dishes. This result indicates that the CD44 expression of passaged cells is not related to cell attachment but rather acts as a marker of mesenchymal and stem/progenitor cells. One explanation for this function is the unstableness of CD44 expression, which easily disappears with maturation^24^. In this study, HPPCs treated with Matrigel, which formed 3D structures with BC-networks, showed a reduction in CD44 expression. Primary cells plated on Matrigel did not proliferate to form SH colonies; however, the sorted cells from SH colonies attached and expanded on Matrigel-coated dishes. Only cells plated on Matrigel-coated dishes could be passaged several times. The passaged CD44^+^ SHs did not require HA for their growth, but HPPCs required Matrigel for maintenance of their self-renewal capability. The Matrigel-dependent expansion and self-renewal of pluripotent stem cells is well established^42^.

Matrigel plays an essential role in the expansion of HPPCs. In addition, it induces the hepatocytic maturation of each cell and the conformational alteration of colonies, which results in hepatic organoid formation. MHs and hepatic stem/progenitors can transdifferentiate into cholangiocytes within a collagen sandwich or gel^43^,^44^. HPPCs did not differentiate into biliary lineage cells in a collagen sandwich culture. However, they did form cystic structures in 3D gels containing collagen and Matrigel. A similar result was reported for Sox9^+^ EpCAM^−^ cells^38^. Primary CD44^+^ SHs and Matrigel-treated HPPCs transplanted in Ret/PH-treated livers were engrafted in bile tubules and differentiated into cholangiocytes. These results suggest that Matrigel components such as laminin-111, Col-IV, heparin sulfate proteoglycans, nidogen, and growth factors^45^ may each play an important role in proliferation, hepatocytic maturation, and transdifferentiation to cholangiocytes. To examine which components of Matrigel are important for cell attachment, we performed preliminarily experiments. In adult healthy rat livers laminin α5 occurs in the space of Disse, whereas laminin α1 does in fetal ones^46^. In addition, laminin α1 is transiently expressed in regenerating livers after PH. As no colony formation was observed in dishes coated with type IV collagen, we examined whether the passaged cells could continue to grow and be passaged by using dishes coated with laminins 111, 511, and 521. As shown in Supplemental Figure S9A, about 17% of the plated cells were attached on laminin-111- or Matrigel-coated dishes, whereas more than 40% of them were on laminin-511/521-coated dishes. At 3 hours after plating, the morphology of the cells on laminin-511/521 was apparently different from that on Matrigel/laminin-111 (Supplemental Figure S9B); the latter retained a spherical shape and the former showed flattened features similar to the progeny that were relatively large cells. In addition, while on laminin-111-coated dishes, similar to Matrigel-coated ones, the passaged cells could form round colonies, the cells on laminin-511/521-coated dishes demonstrated an irregular shape of the cell clusters (Supplemental Figure S9B, a lower plate). Furthermore, as shown in Supplemental Figure S10, the passaged cells on laminin-111 could proliferate to form colonies, which showed typical features of HPPCs, and could be passaged, whereas the cells on laminin-511/521 gradually grew but could not be passaged. These results suggest that the appearance of HPPCs depends on laminin-111, though further studies should be necessary to clarify the role of laminin-111 in the proliferation and maintenance of HPPCs. It is important to elucidate the differences between ECM-based ligands and their receptors in HPPCs and their progeny. Indeed, the progeny continues to possess the capacity of growth and redifferentiation; however, the potential varies among cells and is lower than HPPCs.

In the present study, we showed that HPPCs possess the ability to generate SHs and represent a subpopulation of MHs derived from a healthy adult rat liver. HPPCs can easily expand in chemically defined medium. Although some improvements in the procedure may be required for isolation of human HPPCs, this method generates a large number of healthy hepatocytes from a limited number of donor or surgically resected livers.

## Methods

### Animals

Male F344 rats [dipeptidylpeptidase IV (DPPIV)^+^ strain; Sankyo Lab Service Corporation, Inc., Tokyo, Japan] 6–10 week of age were used for cell isolation. For the transplantation experiment, female F344 rats (DPPIV^−^ strain; Charles River Japan, Yokohama, Japan) were used. All animals were maintained at a constant temperature of 23 ± 1 °C under a 12-h light/dark cycle with standard chow and water ad libitum. The experimental protocol was approved by the Committee on Laboratory Animals and complied with Sapporo Medical University guidelines.

### Isolation, culture, and passages of cells

Primary rat SHs were isolated by the collagenase perfusion method as previously described^22^. Cells were suspended in serum-free Dulbecco’s modified Eagle’s medium/Nutrient Mixture Ham F-12 (DMEM/F12; Sigma-Aldrich Co., St.Louis, MO) supplemented with 20 mM HEPES (Dojindo Laboratories, Kumamoto, Japan), 25 mM NaHCO_3_ (Kanto Chemical Co. Inc., Tokyo, Japan), 30 mg/L L-proline (Sigma-Aldrich Co.), 0.1% bovine serum albumin (BSA; Serologicals Proteons Inc., Kankakee, IL), 10 mM nicotinamide (Sigma-Aldrich Co.), 1 mM ascorbic acid-2 phosphate (Asc2P; Wako Pure Chem. City, Japan), 10^−7^ M dexamethasone (Dex; Wako Pure Chem.), 0.5 mg/L insulin-transferrin-selenium (ITS-X; GIBCO-BRL Invitrogen, Grand Island, NY), 10 ng/mL epidermal growth factor (EGF; BD Biosciences, San Jose, CA) and antibiotics. After the number of viable cells was counted using the trypan blue exclusion test, the cells (2.0 × 10^4^ cells/cm^2^) were plated on 35-, 60- or 100-mm dishes (Corning Inc., Corning, NY) coated with 1 mg/L HA (Sigma-Aldrich Co.). Three hours after plating, the cells were washed with the medium, and the medium was replaced every other day.

### Passages of cells

Nine days after plating, SHs formed colonies consisting of more than 30 cells, which is consistent with our previous report^22^. After washing with PBS, colonies were incubated using 0.02% EDTA/PBS for 5 min. After aspirating the solution, colonies were treated with the separation solution containing 1 mg/mL collagenase (Wako Pure Chem.) and 17,500 U hyaluronidase (Sigma-Aldrich Co.) for 5 min at 37 °C. The cell suspension was collected and stirred for 30 min at 37 °C. The suspension was centrifuged at 150 × *g* for 5 min, and the pellet was resuspended in 2 mM EDTA and 0.5% BSA in PBS. A mouse anti-rat CD44 antibody (BD Bioscience) was added and samples were incubated for 60 min on ice. After washing, the cells were centrifuged at 150× *g* for 5 min. The pellet was resuspended and microbead-conjugated anti-mouse IgG for MACS (Miltenyi Biotec, Bergisch Gladbach, Germany) was added. Magnetic separation was completed using a MidiMACS separation unit, and the positive fraction was collected. The cell fraction was centrifuged at 150 × *g* for 5 min. After the number of viable cells was counted, the cells were plated on 35-, 60- or 100-mm dishes (Corning Inc., Corning, NY) coated with ECMs and cultured in modified DMEM/F12 medium. For passages of epithelial cell colonies, the cells were washed with PBS, incubated with PBS containing 0.01% EDTA and 0.025% Trypsin (Sigma-Aldrich) for 10 min and then detached by pipetting. The cell suspension was centrifuged at 150 × *g* for 5 min. After the number of viable cells was counted, the cells (2.0 × 10^4^ cells/cm^2^) were plated on 35-, 60- or 100-mm dishes coated with 0.2 mg protein/mL (1 mL/35-mm, 2 mL/60-mm, 5 mL/100-mm dish) Matrigel^®^ (Growth factor-reduced, BD Bioscience). In the experiments using laminins the second-passage cells were plated on 12-well plates coated with laminin-111 (10 μg/well; BD Bioscience), −511 (2.5 μg/well; iMatrix-511, Nippi Inc., Tokyo, Japan), and −521 (2.5 μg/well; Biolamina, Stockholm, Sweden). The medium was replaced every other day, and the cells were passaged every 4 weeks.

### Morphological analyses of cultured cells

Phase-contrast photographs of cells were obtained 1, 4, 7, 14, 21, and 28 days after plating using a phase-contrast microscope equipped with a CCD camera (Olympus Optical Co., Tokyo, Japan). The growth of some colonies was pursued and digitally recorded. At least 10 fields per dish were randomly selected and 3 dishes or wells were used per experiment. At least three independent experiments were completed. All captured images were processed using specialized software (Olympus cellSens Dimension Desktop 1.12). The number of colonies, the number of cells per colony, and the size of the cells and colonies were measured.

### Histological Analysis

The animals that underwent cell transplantation were euthanized 30 days after surgery. Livers were removed and immediately cut into 5-mm-thick slices. Liver sections were fixed in 4% paraformaldehyde (Merck KGaA, Darmstadt, Germany) in PBS. Some sections were embedded in Tissue-Tek (Sakura Finetechnical Co., Tokyo, Japan), frozen using isopentane/dry ice, and kept at −80 °C until use. The frozen sections were used for enzyme-based histochemical or immunohistochemical examinations.

Frozen liver sections with a thickness of 7 μm were prepared and air-dried. We used the ABC method for immunohistochemistry. Deparaffinized sections were treated with 0.6% H_2_O_2_ in methanol for 30 min to suppress endogenous peroxidase activity. After blocking with BlockAce for at least 30 min at 4 °C, the slices were incubated with the primary antibody (Supplemental Table S1) for 60 min, rinsed with PBS, and then incubated with a secondary antibody for 30 min. Next, the sections were incubated with an avidin-conjugated biotinylated antibody (VECTASTAIN ABC kit; Funakoshi, Osaka, Japan). 3,3′-diaminobenzidine tetrahydrochloride (DAB) was used as a substrate. Nuclei were counterstained with hematoxylin. Images were obtained using a Zeiss 780 confocal microscope (Carl Zeiss, Oberkochen, Germany) and a confocal laser microscope (Olympus, Tokyo, Japan).

### Fluorescent immunocytochemistry

Antibodies are listed in Supplemental Table S1. The cells were fixed in cold absolute ethanol for 15 min and used for immunocytochemistry. After blocking with BlockAce (DS Pharma Biomedical, Osaka, Japan) for 30 min at RT, the cells were incubated with a primary antibody for 60 min. Dishes were rinsed with PBS and subsequently incubated with Alexa^488^-, Alexa^555^-, or Alexa^594^-conjugated secondary antibodies (Molecular Probe, Eugene, OR) for 30 min. Cells were embedded with 90% glycerol with 0.01% *p*-phenylenediamine and 4,6-diamidino-2-phenylindole (DAPI). To measure the labeling index (LI), 40 μM of 5-bromo-2′-deoxyuridine (BrdU) was added to the medium 18 h before fixation.

### Comprehensive gene analysis and PCR

Differences in the expression profiles of cells were analyzed using an oligo microarray spotted with 30,584 probes (SurePrint G3 Rat Gene Expression v2 G4853B, Agilent Technologies, Santa Clara, CA). The primers used for qPCR are listed in Supplemental Table S2. G3PDH was used for the normalization of relative expression. All DNA microarray data were registered in GEO database (Accession No. GSE 86523). Total RNA extracted from cultured cells was used for cDNA synthesis. To examine the gene expression of colony-forming cells, cloning rings were used to isolate total RNA. RNA was reverse transcribed using an OmniScript RT kit (Qiagen, Hilden, Germany) and random hexamers. Quantitative RT-PCR (qPCR) analyses were completed with TaqMan Gene Expression Assays (Applied Biosystems, Foster City, CA). PCR was performed in triplicate for all of the samples in 96-well optical plates using an ABI Prism 7500 (Applied Biosystems). For reverse-transcription PCR (RT-PCR), genomic DNA was extracted using a GenElute Mammalian Genomic DNA kit (Sigma-Aldrich). TaKaRa Taq PCR kits and a TaKaRa thermal cycler (Takara Bio Inc., Shiga, Japan) were used for all PCR reactions. G3PDH was used for the normalization of relative expression.

### Transmission electron micrographs

Second passage cells cultured for 14 days were fixed in 2.5% glutaraldehyde in 0.1 M cacodylate buffer (pH 7.4) at RT for 60 min, and then postfixed in 1% osmium tetroxide and 1.5% potassium ferrocyanide in the buffer for 2 h. The cells were dehydrated and embedded *in situ* in Epon 812. Ultrathin sections were stained with uranyl acetate followed by lead citrate and examined at 80 KV with a transmission electron microscope (JEOL JEM1400, Tokyo, Japan).

### Flow cytometry

The ploidy of cells was analyzed by flow cytometry. Cultured cells were detached from a dish using PBS containing 0.025% Trypsin and 0.01% EDTA. Cells were fixed in 70% ethanol at −20 °C and then washed with PBS containing 2% FBS. The cell suspension was centrifuged at 200× *g* for 5 min twice. The pellet was suspended in PBS containing 2% FBS and propidium iodide (PI; Dojindo Laboratory) and then filtered through a 35-μm cell strainer (Corning Inc.). Cells were analyzed on a FACS Canto II Flow Cytometer (BD Biosciences). At least 4 × 10^4^ events were recorded for each analysis. Data were analyzed using Kaluza Flow Cytometry Software version 1.1 (Beckman Coulter, Inc., Brea, CA).

### Treatment with fluorescein diacetate

As previously reported^27^, fluorescein diacetate (FD; Sigma-Aldrich Co.) was dissolved in DMSO, and the solution was diluted with culture medium. Next, 0.25% FD was added to the medium, and the dish was rinsed three times with warm PBS. Fluorescent images were immediately recorded using a phase-contrast microscope equipped with a fluorescence device (Olympus).

### Cell transplantation

Recipient rats were given two intraperitoneal injections of Ret (30 mg/kg body weight; Sigma-Aldrich Co.) 2 weeks apart. Four weeks after the last injection, PH was performed (Ret/PH-treated liver). The sorted CD44^+^ cells (5 × 10^5^ cells/0.1 mL PBS) and the cells separated from week 3 of the second passage were cultured with or without Matrigel for 1 week and transplanted into Ret/PH-treated livers via the spleen. Thirty days after transplantation, the animals were euthanized, and the liver was removed to determine if donor cells engrafted.

### Statistical analysis

All data were analyzed using the Turkey-Kramer multiple comparison test. The level of statistical significance was p < 0.05. Experimental results are expressed as the geometric mean ± standard deviation.

## Additional Information

**How to cite this article:** Ishii, M. *et al*. Hepatocytic parental progenitor cells of rat small hepatocytes maintain self-renewal capability after long-term culture. *Sci. Rep.* **7**, 46177; doi: 10.1038/srep46177 (2017).

**Publisher's note:** Springer Nature remains neutral with regard to jurisdictional claims in published maps and institutional affiliations.

## Supplementary Material

Supplemental Information

## Figures and Tables

**Table 1:** Number of colonies used for estimating cell divisions of HPPCs.

Passages	No. of colonies (upper 10%)	Estimated cell population at day 28	Eqaution for estimated cell division	No. of division (periods: days)	Frequency of division
Primary				6 (9)	1/1.5 days
1^st^	33	y = 0.7844e^0.3432x^	y = 0.4952x − 0.3503	13.5 (28)	1/2.07 days
2^nd^	24	y = 0.8869e^0.3194x^	y = 0.4606x − 0.1699	12.7 (28)	1/2.20 days
3^rd^	24	y = 0.9944e^0.2819x^	y = 0.4065x − 0.0047	11.0 (28)	1/2.54 days
4^th^	14	y = 1.253e^0.2174x^	y = 0.3137x − 0.3395	9.0 (28)	1/3.11 days
