# Supplementary material for: Hepatocytic parental progenitor cells of rat small hepatocytes maintain self-renewal capability after long-term culture
Source: Sci Rep. 2017 Apr 11;7:46177. doi: 10.1038/srep46177 (PMC5387414; doi:10.1038/srep46177)
Supplement: Supplemental Information [file srep46177-s1.pdf]

Hepatocytic parental progenitor cells of rat small hepatocytes maintain self-renewal capability after long-term culture

Masayuki Ishii, Jun-ichi Kino, Norihisa Ichinohe, Naoki Tanimizu, Takafumi Ninomiya, Hiromu Suzuki, Toru Mizuguchi, Koichi Hirata, Toshihiro Mitaka.

Supplemental Figure Legends

Supplemental Figure S1

Matrigel dependency of CD44 SH growth. Matrigel® (10 mg/mL) was diluted into the culture medium. Colonies with more than 10 cells were counted.

Supplemental Figure S2

The ploidy of the first-passaged $CD44^+$ SHs cultured for 28 days was examined by flow cytometry. The fraction of single cells was separated from the fraction of cells with dense cytoplasm. Diploid (2n), tetraploid (4n), and octaploid (8n) cells are shown.

Supplemental Figure S3

Experimental design of cell passages.

Supplemental Figure S4

Phase-contrast photographs of typical colonies showing various types of morphology in each passage at 14 (1–3) and 21 days (4–9) after plating. Photos in each passage (1–3) show the typical morphology of colonies with high growth activity. The colonies have a relatively round shape, and contain homogeneously small-sized, polygonal, and mononuclear cells. The size of each cell gradually increases with each passage. Over time, colonies with irregular shapes that consist of cells with a relatively large cytoplasm (4–9) appear in culture. These colonies include some binucleate cells, but cells with multinuclei were rarely observed. Scale bars = 100 μm.

Supplemental Figure S5

Reproducibility of colony formation was confirmed. Three independent experiments were performed, and the colony-formation capability of $CD44^+$ SHs was analyzed by counting the number of cells after 14 days in culture. The distribution of colony sizes was not different among experiments up to the second passage, but a difference in the number of colonies with high growth activity appeared after the third passage.

Supplemental Figure S6

(A) Based on the data shown in (B), a formula of exponential approximation was calculated to estimate the number of divisions within 28 days for each passage. (B) To establish linear equations for each passage, the mathematical expression was converted to a logarithmic scale.

The average number of cell divisions over 28 days is 13.5, 12.7, 11.0, and 9.1 in the first, second, third, and fourth passages, respectively.

Supplemental Figure S7

Expression of *Alb, Hnf4α, C/ebpα, CK19*, and *CD44* genes was examined by qPCR. At passage, total RNA was extracted from isolated cells.

Supplemental Figure S8

Incorporation of donor cells into small bile ductules in Ret/PH-treated rat livers. Fluorescent immunohistochemistry for dipeptidyl peptidase IV (DPPIV) and cytokeratin 19 (CK19) was examined. The sorted $CD44^+$ SHs (CD44) and the second passage cells cultured for 21 days without (third SHs) or with Matrigel (third SHs+MG) were transplanted. The livers were removed at 21 days after transplantation and fixed. Arrows (yellow) show $DPPIV^+$ $CK19^+$ cells.

Supplemental Figure S9

The second-passage cells (1 x10^4 cells/well) were plated on 12-well plates coated with laminin-111, -511, -521, and Matrigel. (A) The rate of attached cells was measured. Three wells were used per experiment. The bars show SD. (B) Phase-contrast photographs of plated cells on each substrate at 3 hrs (upper row, all the same magnification) and 7 days after plating (lower row, all the same magnification).

Supplemental Figure S10

Colony formation of the passaged cells on laminin-111-coated dishes. The phase-contrast photos were taken at 28 days after subculture. The colonies of third-passage cells seemed more compact than those of second-passage cells and the growth rate of the cells may be maintained while HPPCs on Matrigel gradually reduce their proliferative ability after the third passage.

Supplemental Figure S1

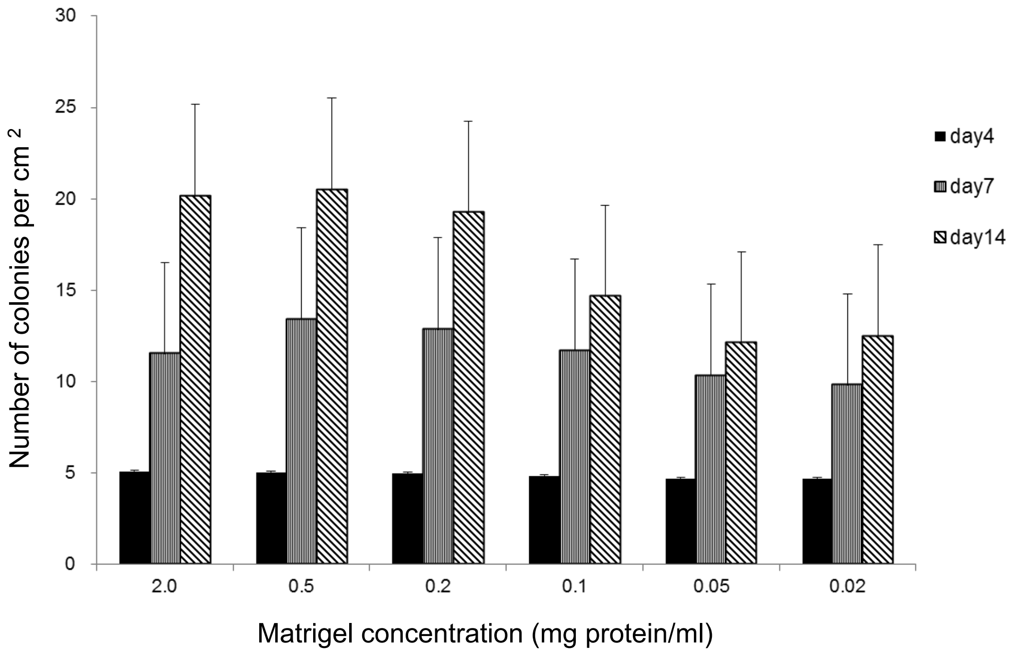

Supplemental Figure S2

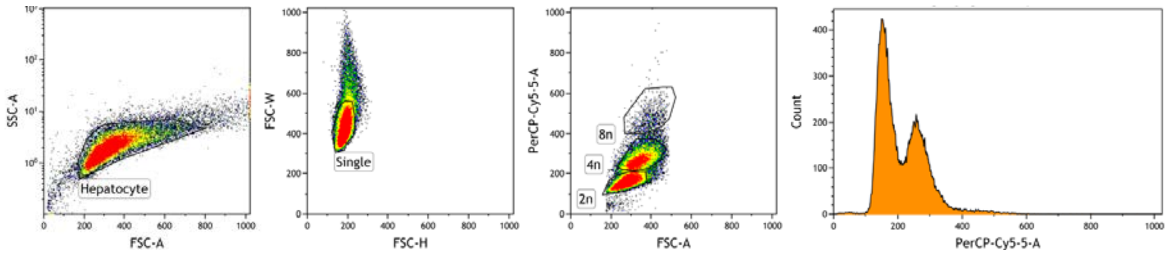

Supplemental Figure S3

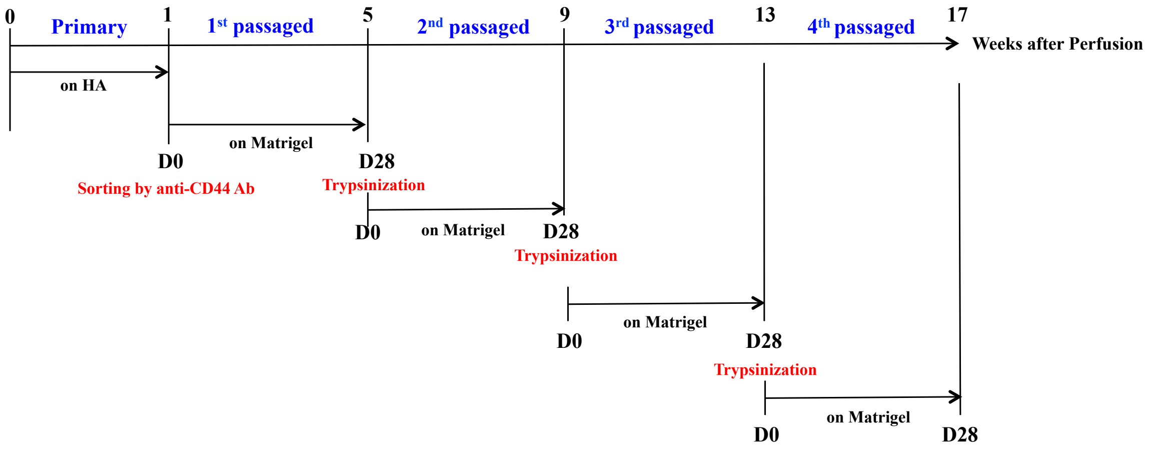

Supplemental Figure S4

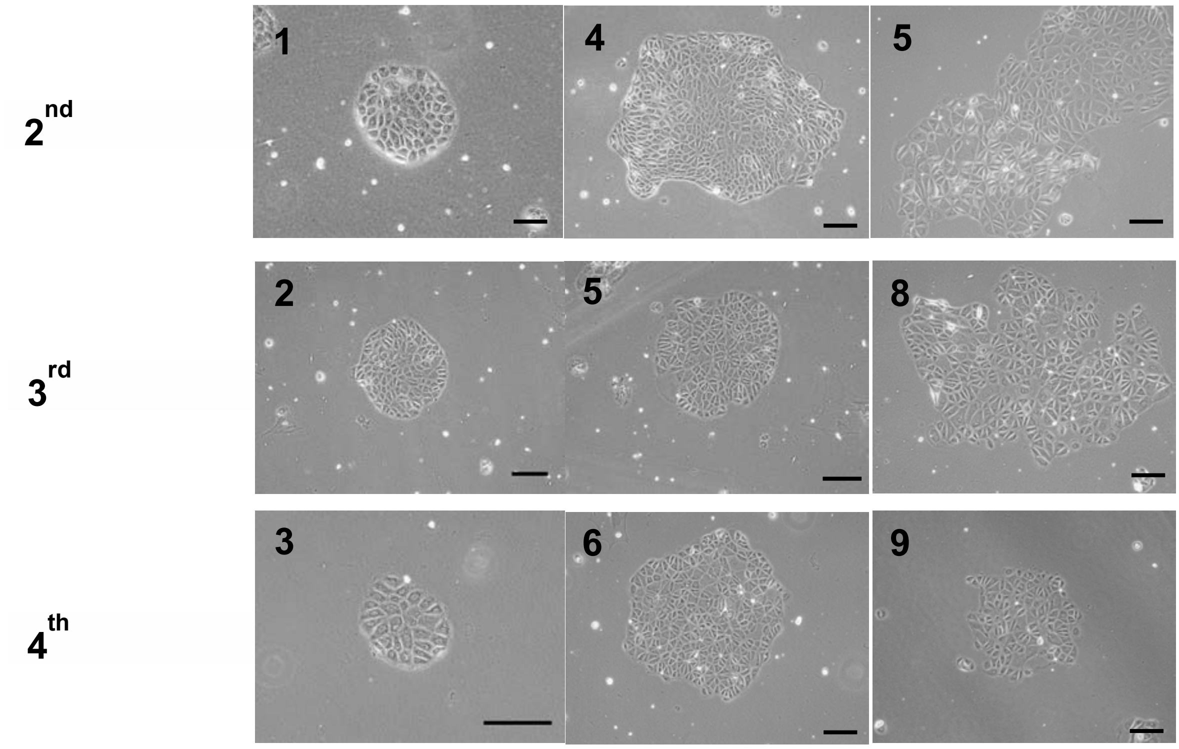

Supplemental Figure S5

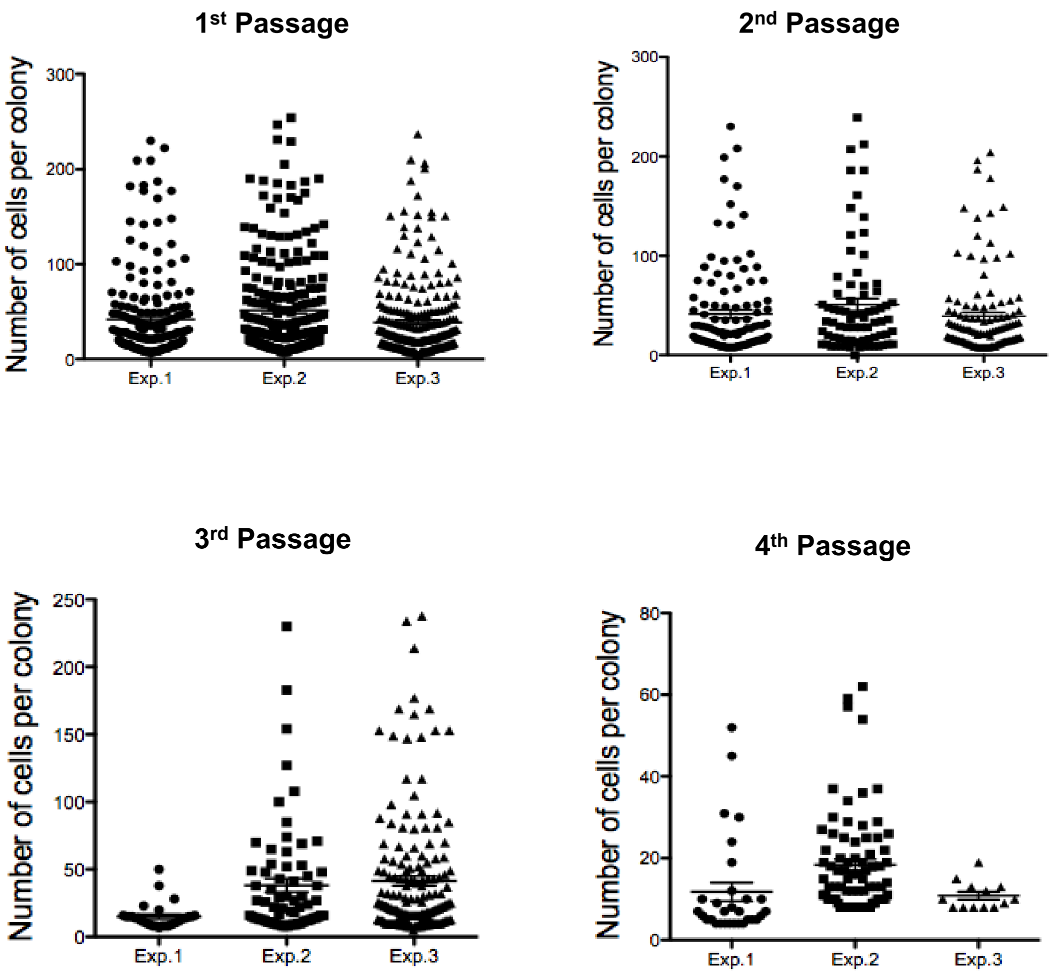

Supplemental Figure S6

A

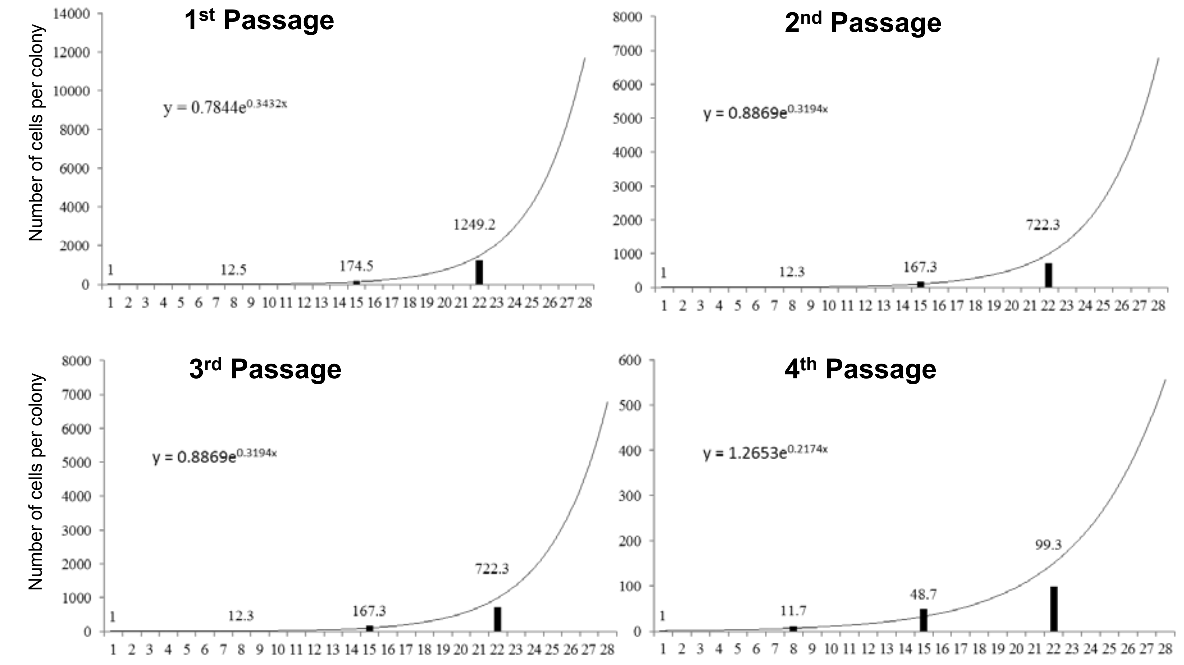

B

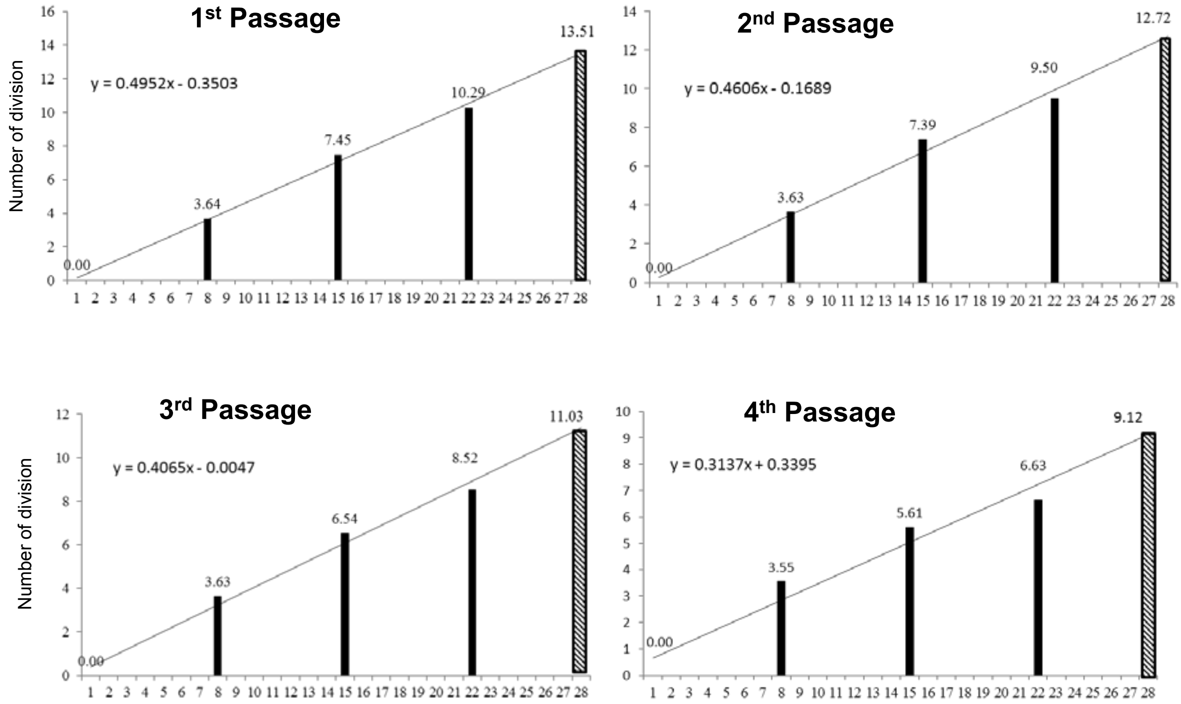

Supplemental Figure S7

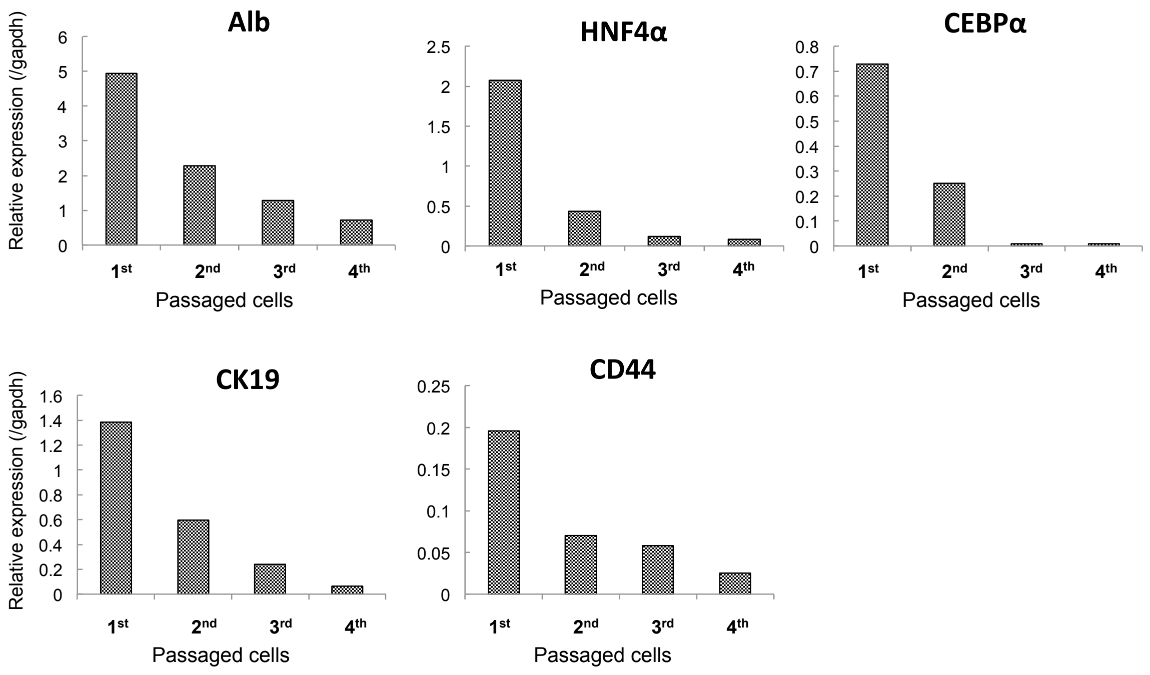

Supplemental Figure S8

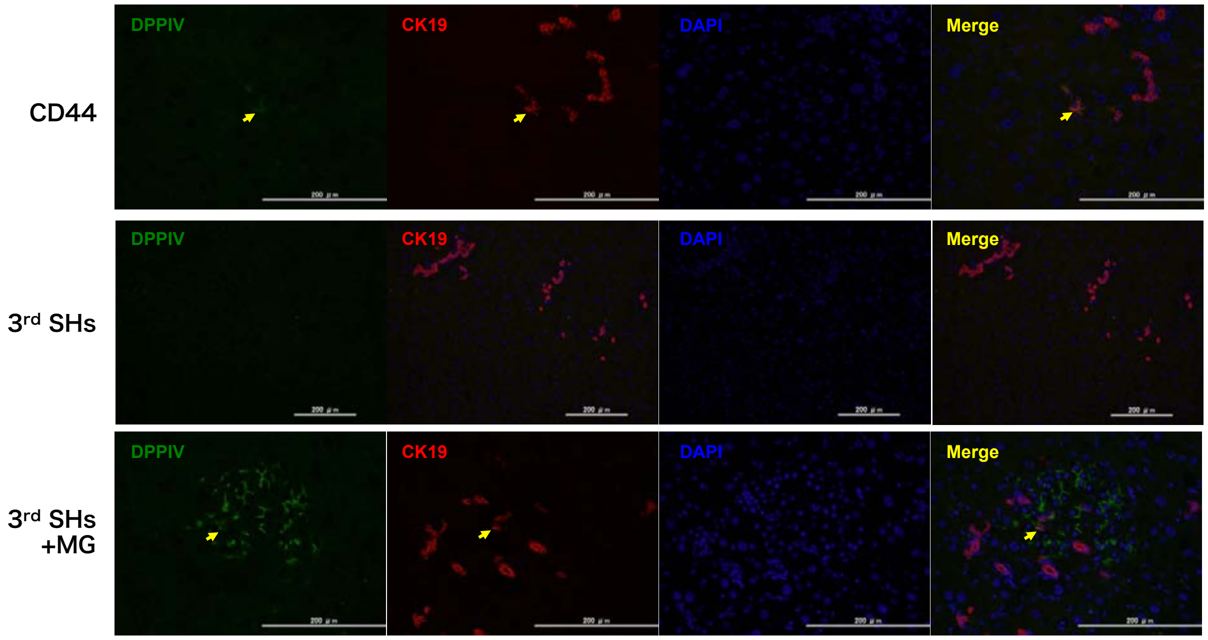

Supplemental Figure S9

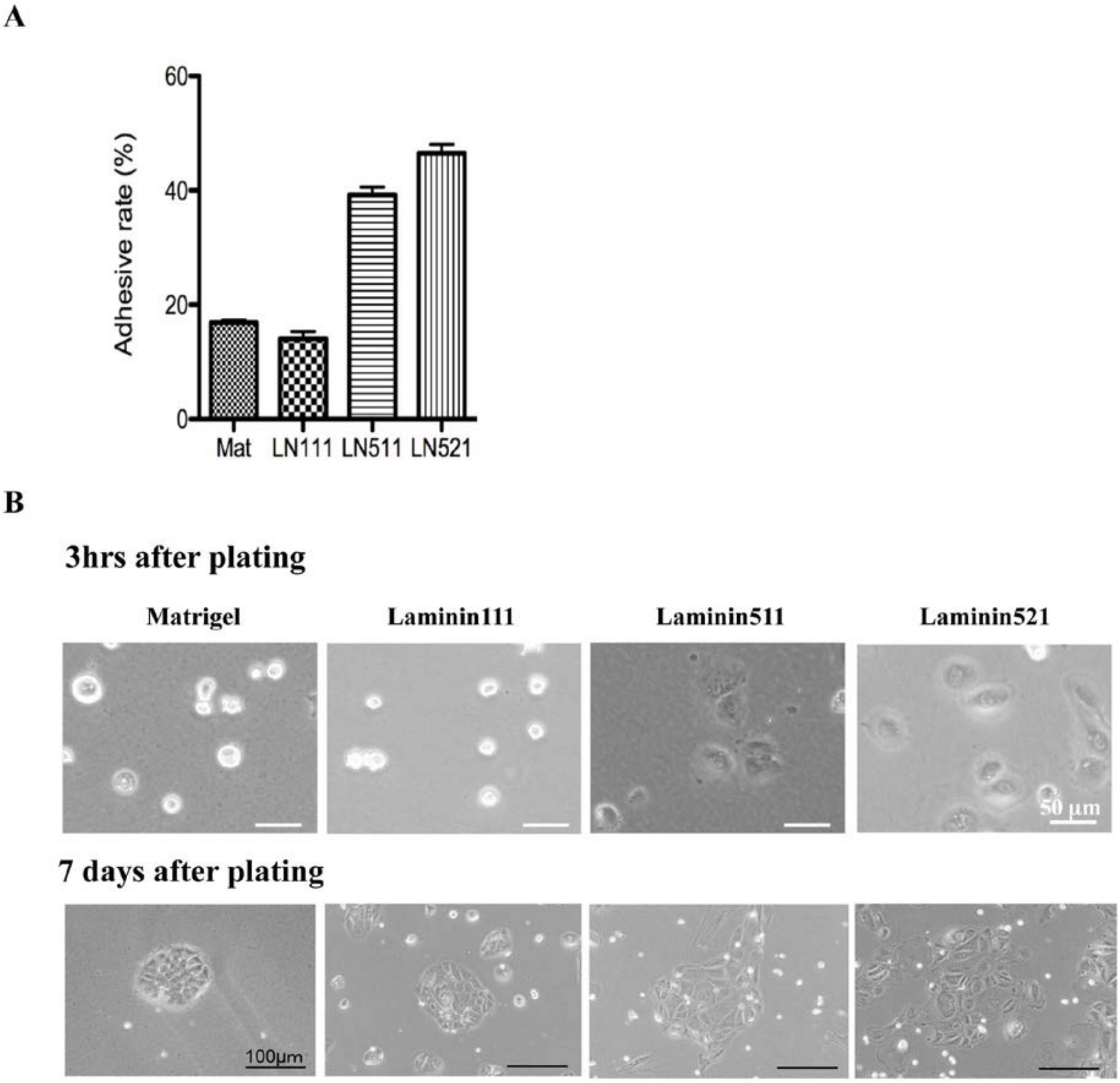

Supplemental Figure S10

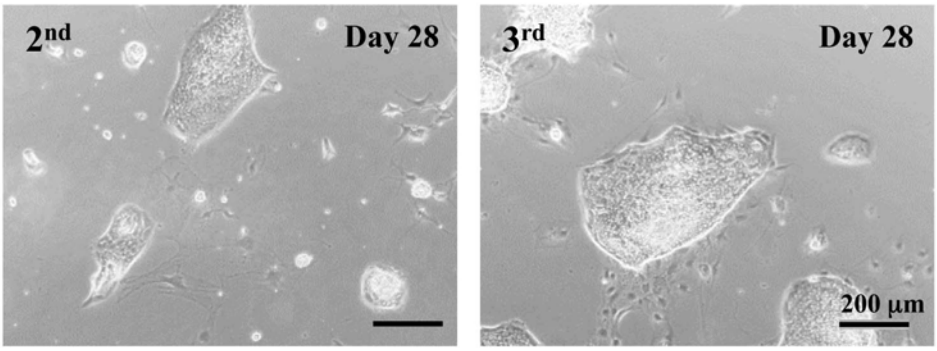

Supplemental Table S1 List of antibodies used in the present experiment

Antibodies	Host	Company of Products	Dilution
CD44 standard form	mouse	BD Biosciences Pharmingen, Franklin Lakes, NJ	1:1000
HNF4α	goat	Santa Cruz, Santa Cruz, CA	1:200
C/EBPα	rabbit	Santa Cruz, Santa Cruz, CA	1:200
BrdU	mouse	DakoCytomation, Glostrup, Denmark	1:200
Albumin	goat	ICN pharmaceuticals, Costa Mesa, CA	1:1000
CYP2B1	goat	Daiichi Kagaku Yakuhin, Tokyo, Japan	1:200
MRP2	mouse	Alexis Biochemicals, San Diego, CA	1:200
BSEP	goat	Santa Cruz, Santa Cruz, CA	1:200
DPPIV	mouse	Santa Cruz, Santa Cruz, CA	1:200
CK19	rabbit	Generous gift from Prof. Atsushi Miyajima	1:500
Alexa Flour 633 Phalloidin	-	Molecular Probes, Eugene, OR	1:300
Mouse IgG2a+b microbeads	rat	Miltenyi Biotec, Bergisch Gladbach, Germany	1:5
Alexa 488-conjugated	donkey	Molecular Probes, Eugene, OR	1:500
Alexa 555-conjugated	donkey	Molecular Probes, Eugene, OR	1:500
Alexa 594-conjugated	donkey	Molecular Probes, Eugene, OR	1:500

Suppplemental Table S2 List of primers used in the present experiment

Genes	Realtime-PCR Primers
Albumin	Rn 01498425_m1
Hnf4a	Rn 00573309_m1
Cebpa	Rn 00560963_s1
Tat	Rn 01640359_m1
Tdo2	Rn 00574499_m1
Cyp2b1	Rn 01457875_m1
CD44s	Rn 00681157_m1
CK19	Rn 01496867_m1
G3PDH	Rn 01775763_s1

Supplemental Table S3 Efficiency of cell attachment and the growth ability of the cells in each passage

Passages	Attached cells at day 1 after plating (%)	Total growth of attached cells at 28 days after passage (fold)
1st	16.90 ± 0.50	11.80 ± 2.17
2nd	15.13 ± 0.12	11.07 ±4.06
3rd	14.87 ± 0.96	4.27 ± 0.31
4th	12.77 ± 0.58	4.4*

Mean ± SEM

* n=2

At every passage the number of attached cells per field were counted at 1 day after plating and the number of the attached cells on a dish was estimated. The expansion rate (fold) of the cells was calculated by the equation that the number of recovered cells was divided by the number of attached cells. At least 3 independent experiments were performed.

Supplemental Table S4 Ploidy pattern of cells analyzed by flow-cytometry

**Cells**	**2n (%)**	**4n (%)**	**8n (%)**
MHs	17.5 ± 2.3	69.3 ± 9.0	13.2 ± 7.4
CD44+ cells	34.4 ± 7.2	35.1 ± 14.5	30.0 ± 11.5
1st passaged cells at day 28	52.1 ± 5.3	44.0 ± 4.6	3.8 ± 0.9

MHs: mature hepatocyte isolated from an adult healthy rat liver

CD44^{+} cells: cells sorted by an anti-CD44 antibody

1st passaged cells: cells cultured for 28 days and then isolated.

Supplemental Table S5 Colony formation of 1st passaged cells in the medium that each constituent is deleted

Deleted substances	No. of colonies counted	No. of cells per colony	Labeling Index (% of BrdU+ cells)
Control	113	35.4 ± 2.3	29.1 ±1.5
Nicotinamide	0	-	-
EGF	45	18.1 ± 1.4*	4.6 ± 0.9*
ITS	70	26.7 ± 2.1*	24.3 ± 1.6
Dexamethasone	79	25.3 ± 1.6*	27.9 ± 2.1
Asc2P	101	32.0 ± 2.6	32.9 ± 2.0

Mean ± SEM

* P<0.05 vs Control
